# What evidence exists on the effect of the main European lowland crop and grassland management practices on biodiversity indicator species groups? a systematic map

**DOI:** 10.1186/s13750-024-00347-0

**Published:** 2024-08-17

**Authors:** Coralie Triquet, Marie Perennes, Robin Séchaud, Markus van der Meer, Yvonne Fabian, Philippe Jeanneret

**Affiliations:** 1 Agroecology and Environment, Agroscope, Reckenholzstrasse 191, CH-8046 Zurich, Switzerland; 2 Swiss Ornithological Institute, Sempach, Switzerland

**Keywords:** Farming practice, Monitoring, Ecological infrastructures, Fertilization, Grazing, Pesticides, Flora, Arthropods, Pollinators, Birds

## Abstract

**Background:**

The intensification of the agricultural practices in Europe over the last decades has drastically transformed the agroecosystems. The simplification of the landscape, the loss of semi-natural habitats and the application of chemicals on crops led to biodiversity decline in agricultural landscapes, raising substantial concerns about the loss of essential ecosystem services, such as pollination or pest control. Depending on the location, the scale and the regional context, different indicator species groups (ISGs) are regularly surveyed to assess the state and trend of biodiversity changes in agroecosystems. Although the high diversity of these ISGs allows assessing different biodiversity aspects (e.g., trophic levels, bio-physical compartments, scale of indication), it complicates the interpretation of the results and thus their practical application. In addition, species diversity metrics are various, from simple species counts to more complex measurements of diversity indices, sometimes with antagonistic responses. Here, to meet the pressing need for synthesis in this complex topic, we follow a standardized systematic map protocol to collect and summarize the literature reporting field evidence of the effects of the main agricultural management practices (AMPs) in arable crops, grasslands and ecological infrastructures on a set of ISGs in European lowland farming areas.

**Methods:**

Searches of literature were made using online publication databases, search engine and specialist websites in English. Gathered publications were screened for relevance following inclusion/exclusion criteria published in a prior protocol. We extracted and mapped information about experimental design, monitoring methods, ISGs and AMPs studied and the diversity measures presented in each included publication. These parameters are structured in available data coding sheets.

**Results:**

The search gathered 20,162 references from which 1208 remained after full text eligibility screening. Main areas studied are in Western Europe, and the number of studies increased exponentially from 1984 to 2022. Most publications are experimental and on-farm studies which assess AMPs effects at the field scale. Main studied AMPs are fertilization, grazing, organic farming, tillage, mowing and herbicide application. Most ISGs used to study their impacts are flora, carabids, spiders, birds, bees and annelids, often combined with other ISGs. The combinations between AMPs and ISGs studied are detailed as well as monitoring methods. The most used diversity measures are abundance, species richness, Shannon index, evenness, and community composition.

**Conclusions:**

We identified several knowledge clusters: (1) organic farming, fertilization, tillage, grazing and mowing impact on a wide range of ISGs, (2) flora response to agricultural practices, (3) annelids response to agronomic interventions that impact soil structure (e.g., tillage, fertilization, crop rotation, crop residue management), (4) butterflies and orthopterans response to mowing and grazing effects in grasslands, (5) the use of bird monitoring for the impact for assessing the efficiency of AES implementation at the landscape scale. We highlight that further research should be conducted on ISGs that are until now poorly studied regarding agricultural practices, such as amphibians, reptiles, gastropods, millipedes and centipedes. More field evidence of the effects of diversification practices such as intercropping, undersowing, intermediate cropping, and agroforestry are needed to draw conclusions on their benefits on biodiversity.

**Supplementary Information:**

The online version contains supplementary material available at 10.1186/s13750-024-00347-0.

## Background

Agriculture is the most abundant land use in Europe, covering approximately 45% of the total land area of the EU-27 [1]. The intensification of agricultural practices over the last decades has profoundly modified the functioning of agroecosystems and threatened its biodiversity, resulting in an unfavorable conservation status for 76% of agricultural habitats and 70% of their inhabiting species [2, 3]. The drivers of biodiversity loss are diverse, but the main ones are the simplification and homogenization of the landscape, the loss of semi-natural habitats and the increased application of fertilizers and pesticides on fields [4–8]. The decline of biodiversity in agroecosystems raises considerable concerns about the deficiency of ecosystem services essential for agricultural productivity [9–11], such as pollination, habitat maintenance, formation of soils and pest regulation. The protection of biodiversity and associated ecosystem services is thus a crucial step to ensure the long-term sustainability of farming systems. Assessing the state and trend of biodiversity in agricultural land is a major challenge, especially given the variety of agricultural management practices (AMPs) and the difficulty of choosing indicator species groups (ISGs) that are ecologically meaningful and representative of biodiversity [8, 12–16]. Currently, only birds and butterflies are monitored in agricultural areas at the European scale, both showing substantial declines over the past decades [17, 18]. There are, however, numerous other ISGs monitored in national programs or for specific research projects [19–21], and their use depends on the scale considered, the specific context and the objectives of the projects [15]. Similarly, while the most common quantitative metric of an ISG diversity is its species richness (number of species), more complex measurements of species heterogeneity (i.e., species evenness, or Shannon index) are often used, sometimes revealing different trends [22, 23]. Consequently, the large number of ISGs, the numerous methods for monitoring them, and the various types of diversity responses measured make their utilization and their interpretability more complex, highlighting the pressing need for synthesis in this topic. In the first instance, an overview would help to identify knowledge gaps. Further, matching and discrepancies between responses of ISGs and their measurement options (diversity indices, community analysis) would reveal very useful synergies and tradeoffs that would have to be taken into account by setting up biodiversity-friendly practices and wider conservation actions.

The aim of this systematic map is to gather and describe the literature documenting the effects of the main European lowland AMPs on ISGs and report them following the systematic evidence synthesis standards (ROSES checklist provided in Additional file 1). We considered the most frequent farming practices grouped into 10 main categories of AMPs of lowland agriculture in Europe: tillage, fertilization, sowing, irrigation, crop protection, harvesting/mowing/grazing, cover crops and intercropping, rotation, ecological infrastructure implementation, and Agri Environmental Schemes (AES) adoption (including organic agriculture) (see inclusion/exclusion criteria grid in Additional file 3). We selected ISGs that cover a wide range of trophic levels and ecological niches, and that are known and used as indicator in biodiversity conservation and provision of ecosystem services [19], resulting in a set of 24 candidates: flora, mammals, birds, reptiles, amphibians, spiders, bees, parasitoid wasps (ichneumonids and braconids), orthopterans, butterflies, carabids, coccinellids, staphylinids, syrphids, lacewings, ants, slugs, snails, annelids, nematodes, soil mites, springtails, millipedes, and centipedes (Fig. 6; Additional file 3). We made a focus on the monitoring methods and the diversity measures used for their assessment. We restricted the synthesis on the main types of agricultural fields present in Europe, grouped in three main categories (annual crops, grasslands, and ecological infrastructures; Additional file 3), excluding perennial crops (orchards and vineyards, see methods section, under deviation from the protocol) [25]. The present systematic map aims to help prioritizing future scientific research by identifying knowledge gaps, as well as providing a synthesis of knowledge for stakeholders in the field and providing tools for decision makers to evolve toward more sustainable agriculture.

## Objectives of the map

The goal of this systematic map is to estimate the current state of knowledge regarding the effects of the main European lowland AMPs on biodiversity. Our main objectives are threefold: (1) to report the evidence of the effects AMPs on different ISGs, (2) to record the different metrics of species diversity used and (3) to identify the monitoring methods used, while being particularly attentive to the emergence of novel techniques such as the use of drones or genetic identification methods. Together, these three objectives allow to assess the current research state on the topic, to provide guidance in selecting ISGs and appropriate measurement methods, and to identify knowledge gaps that warrant further research.

### Primary question

What evidence exists on the effect of the main European lowland crop and grassland management practices on biodiversity indicator species groups?

### Components of the primary question

Based on the PICO framework [24], which enables to define a research question based on four main themes (Population, Intervention, Comparator and Outcome), the primary question components are:

- Population (P): the biodiversity indicator species groups (ISGs)
- Intervention (I): the European lowland arable crop and grassland management practices (AMPs)
- Comparator (C): the comparison before/after AMP interventions, between AMPs and controls, or between different AMPs
- Outcome (O): measure of change of the ISGs (i.e., abundance, diversity measures, evenness, species and ecological traits composition)

### Secondary questions

Five secondary questions are addressed in this systematic map.

1. What are the most surveyed ISGs? Are there temporal variations?
2. What are the main ISGs monitoring methods? Are there trends towards a change in monitoring methods for some ISGs?
3. What types of diversity measurements (i.e., structural, or functional biodiversity measurements) are most often reported?
4. Are the ISGs generally surveyed alone or combined? Which combinations are the most frequent?

## Methods

### Deviations from the protocol

#### ISGs

We did not change the selected ISGs list presented in the prior protocol [34]. As the ISG “Coleopterans” in the initial ISG list was restricted to the families coccinellids, carabids and staphylinids, the effects on those are reported separately.

#### Definition of AMP categories

AMP categories were slightly modified to include a wider range of AMPs:

- Tillage, including all soil preparation interventions and their variations.
- Sowing, including all management practices regarding sowing and plantation.
- Fertilization, including all organic and mineral fertilization strategies.
- Irrigation.
- Crop protection, including mechanical weeding and use of herbicides, insecticides, molluscicides, rodenticides, fungicides, and biocontrol agents.
- Harvesting/Mowing/Grazing, and their variations, depending on the crop type.
- Intercropping, intermediate cropping and all other diversification practices incorporating a cover crop such as undersowing.
- Crop rotation, i.e., the sequence in which different crops are occurring in time, in opposition to monoculture.
- Ecological infrastructures implementation, containing hedges, vegetation strips, fallows/set-asides, flower fields, and special structures such as ditches and ponds.
- AES adoption, including organic agriculture and other sustainable production methods that involve combinations of practices.

#### Field type categories

In the protocol, the types of fields under investigation encompassed various perennial crops, such as vineyards, apple, pear, apricot, cherry, and plum orchards. However, AMPs search terms did not sufficiently reflect the agricultural practices specific to vineyard and orchard cropping systems which would need a particular searching process. Indeed, these systems often involve a large number of manual practices not typically used in arable cropping, resulting in a limited extraction of articles relevant to perennial crops. Given the broad scope of the systematic map and the existence of a comprehensive systematic map focusing on the effects of agricultural practices on ISGs in orchards [29], we opted for exclusion of perennial crops from the field types of interest and concentrate on arable crops, grasslands, and ecological infrastructures. Ecological infrastructures (EI) are thus defined as a field type of interest, but their implementation can also be considered as AMPs. We included articles studying the effect of EI implementation on ISGs on adjacent grassland and arable crops, or compared with a control representing the situation before/without the EI. We also included articles studying the effect of EI management, e.g., mowing, trimming, fertilization, method of implementation or seed mixtures used.

#### Study validity assessment

As planned, we extracted study characteristics that give a reliable overview of the study relevance and validity of each study. However, we did not combine them to obtain a unique study validity score (value of fit with the study question) as initially planned, because of the wide diversity of study designs that are nevertheless valid. This way we rather highlighted deficiencies that should be taken into account for further quantitative syntheses (see section “Article screening and study eligibility criteria”).

### Search for articles

#### Definition of search terms, reference searches and limits of the search scope

**Fig. 1:**
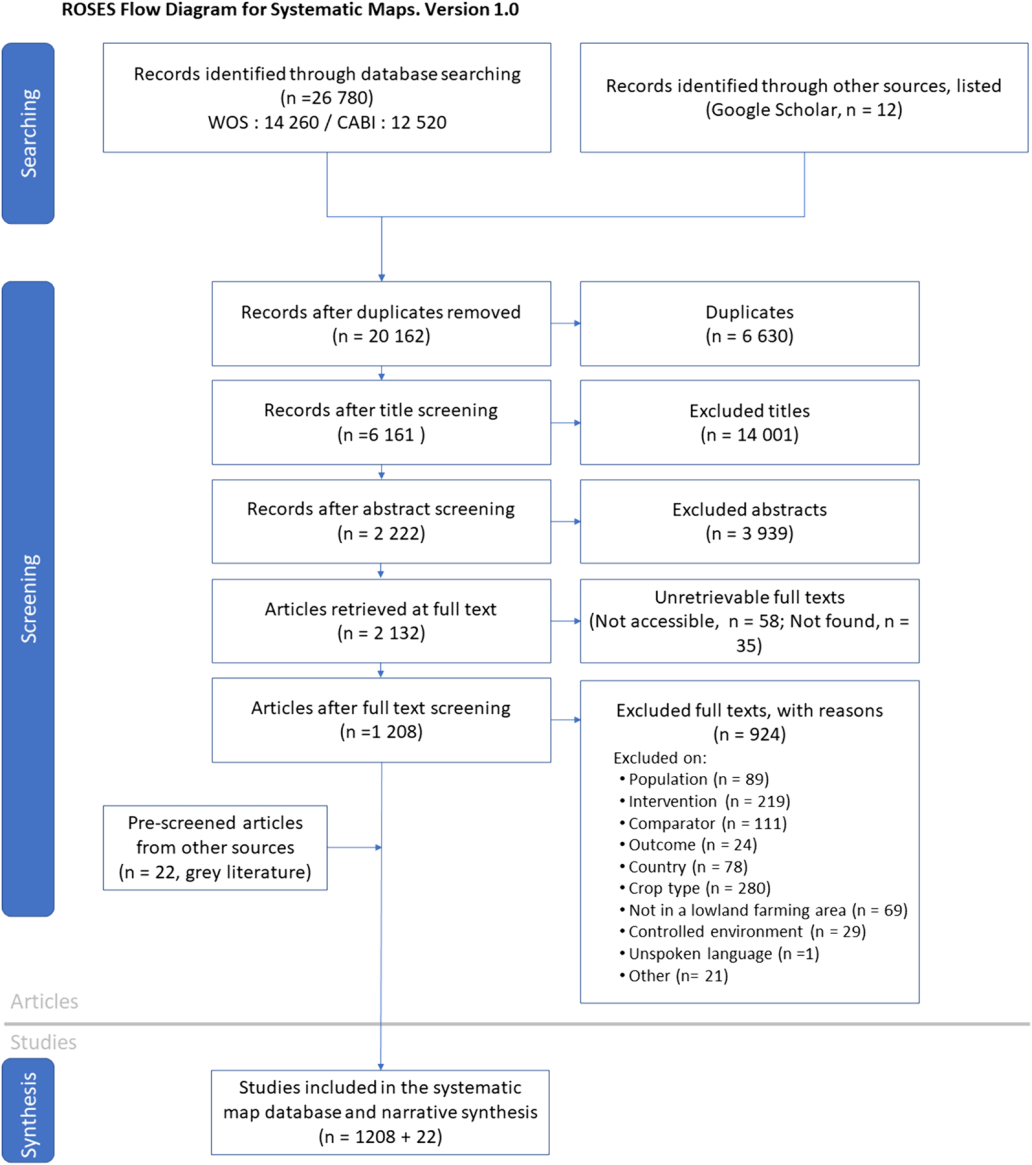
ROSES flow diagram illustrating the literature search and screening process [35]

We relied on different reviews, meta-analyses, books, reports or scientific articles (see for example [8, 13–15, 19, 26–30]) to develop a list of ISGs used in various research domains such as nature conservation, ecosystem functionality, biodiversity indicators or ecosystem services. The 24 identified ISGs are intended to cover a wide range of ecological niches and trophic levels (see Fig. 1 from protocol [29]. To collect the literature corresponding to this list, we developed a set of 93 search terms (search strings are shown in Additional file 2). Although soil microorganisms including fungi, bacteria, and archaea, are crucial components of biodiversity for agriculture with many implications for ecosystem services, we excluded them as ISGs because we considered them beyond the scope of the study with up to 50 000 references that would require a map in itself. AMPs in European arable systems are spatially and temporally diverse, we thus chose to group them into broader categories, and built a set of 55 search terms to effectively gather relevant literature (Additional file 2; see interventions in Fig. 5). Concerning the outcomes, we were interested in studies reporting a difference or a change in the abundance, diversity, or community composition of the ISGs. We combined 11 search terms to include taxonomic, structural, and functional diversity indices. Each of these terms was then associated with the word “species” (i.e., “species richness”) or with one of the ISG search terms (i.e., “spider richness”). When the search platform offered the possibility of using proximity operators, they were combined with the Boolean operator “NEAR/3” (in Web of Science) to find records where both terms are within three words of each other (i.e., “richness of spiders”), otherwise they were combined with “AND”. Finally, to restrict the literature search to European agricultural environments, we defined two additional sets of keywords. Six “Environment” keywords aimed at focusing on agricultural landscapes (crop and grassland), and 49 “Location” keywords restricted the search to European countries. The geographical range of the study includes most continental Europe, apart from Russia and Turkey (and countries further east of the latter), and islands, as those are commonly known to have different conditions from the continent (i.e., species guilds, types of agriculture or weather conditions). Even if we focused on three main field type categories (annual crops, grasslands, and ecological infrastructures), we did not define specific keywords to select for field types during the literature search phase, but we used them as inclusion/exclusion criteria during the screening process (see Additional file 3). The same logic was applied to include only lowland agricultural areas: mountains, uplands, forest and coasts were in the list of exclusion criteria.

Search terms within categories were combined using the Boolean operator “OR”, and between categories using the Boolean operator “AND” (all search strings used can be found in Additional file 2). This implies that studies must include at least one term of each of the five categories to be retained. When possible, the search was restricted to the article title, abstract and keywords, except for the “Location” search terms that was screened across the full text. Due to keyword number limitations in the searches, we conducted a literature search separately for each ISG. This means that the keywords for the categories Intervention, Outcome, Environment and Location were combined to each ISG keyword successively.

We searched for relevant literature on the Web of Science Core Collection and CABI platforms using the institutional access of Agroscope. Extraction of references was made the 19th of April 2022. We additionally conducted an internet search on the Google Scholar website using a simplified search string (Additional file 2) at the whole text level (as it is not possible to restrict the search fields in Google Scholar). The first 500 results of google scholar were exported to an Excel format and screened. To reduce the algorithm biases associated with previous internet searches, browser history and cookies were disabled during the internet search and the “private” navigation mode was used.

A comprehensive search for grey literature at the European scale would go beyond the scope and resources of this systematic map. Nevertheless, supplementary searches were still carried out for Switzerland where access has been realistically possible. We then searched for grey literature in English and French on Swiss specialized websites (see Additional file 2). A small number of documents fitting the inclusion criteria grid were found. Thus, the final analyses were conducted without the grey literature to avoid any grey-literature-based bias in the conclusions, but the documents were also included in the database.

#### Comprehensiveness of the search

To evaluate the comprehensiveness of the literature search, we compared different search strings results with a test-list of articles considered to be relevant. To produce the test list, we first selected 60 articles based on our knowledge of the literature, that covered the various aspects of the systematic map and expected in the literature search results (i.e. articles that fit with the topic and respect our inclusion criteria). Secondly, to ensure the diversity and representativeness of the test-list, we added pertinent literature cited in five key publications on biodiversity in agriculture: a review on the biodiversity in agricultural areas [12], a review of soil biodiversity [14], a European project on agricultural biodiversity [31], and two comprehensive research articles on Swiss biodiversity in agriculture [32, 33]. This resulted in a test-list of 90 articles (list given in Additional file 2), published over a period of 30 years (from 1991 to 2021) in 39 different journals. We extracted 87 from the 90 articles of the test-list (96.7%) with the search strings used for this work.

### Article screening and study eligibility criteria

#### Screening process

First, as the different search sources partly reported the same references, duplicates were removed based on the DOI identifier, and on title for references without DOI. Then, the study screening process has been successively performed at the title, abstract and full-text levels. At each level, articles were classified as eligible (included in the review) or ineligible (excluded from the review), or uncertain. In the latter case, articles were passed to the next level of screening and reevaluated (i.e., articles uncertain at the title screening level were passed to the abstract screening stage). For each rejected article, we recorded the level (title, abstract, full text) and the reason (list of choices) of exclusion, which is available in Additional file 6.

Full articles were obtained from the literature access of Agroscope, yielding a very good accessibility to full texts (97.4% of full text were accessible, only 58 articles were not accessible). To guide reviewers’ choices of including or excluding an article, we defined a set of criteria (Additional file 3), and assessed the replicability of the screening process by comparing the choices made by the reviewers (RS, CT, and MvdM for title and abstract screening, CT and MP for full text screening). To do so, a subset of 150 articles were screened independently by two reviewers, and their agreement compared and evaluated using Cohen’s kappa coefficient (k > 0.6 considered as consistent) at each screening level. In case of inconsistency (k < 0.6), the reviewers discussed to resolve the reasons of inconsistency in their choices, clarified or adapted the criteria, and then screened a new subset of articles until consistency was reached. When consistency was sufficient (k > 0.6), reviewers still discussed and solved the remaining disagreements to ensure a high replicability.

#### Eligibility criteria

To be included in the systematic map, articles needed to fulfill nine conditions:

- Eligible population: articles had to include at least one of the ISGs.
- Eligible intervention: articles had to include at least one of AMPs.
- Eligible comparator: articles had to compare ISGs before/after intervention, between an intervention and a control, or between different interventions.
- Eligible outcome: articles had to report a measure of species diversity (e.g., richness, abundance, or evenness).
- Eligible environment: articles had to report research conducted in lowland agricultural landscapes.
- Eligible location: articles had to report studies conducted on the European mainland.
- Eligible study design: articles had to report and analyze monitoring or experimental field-data.
- Eligible field type: articles had to report the effects of an AMP on an ISG in one of the crop types considered in the present study (arable crops, grasslands, and ecological infrastructures).
- Eligible language: articles had to be written in English. Grey literature in French was also accepted.

We excluded all articles not accessible as full text and those that only addressed:

- One-time events (e.g., a unique pollution event), as well as studies which do not directly study the effects of AMPs on ISGs (e.g., bird population fluctuation over time in agricultural areas, without specific AMPs associated).
- Studies reporting ISGs without further comparison were excluded (e.g., state of the bee population visiting a flower strip).
- Studies conducted in non-lowland farming landscapes (i.e., forests, coasts, upland or alpine environments). We also discarded wetland (i.e., fen, flooded areas) because of their very specific conditions leading to particular practices.
- Studies conducted outside the geographical area under consideration.
- Modeling papers, books, reviews, and meta-analyses were excluded, as well as experiments conducted in controlled conditions (i.e., laboratory, pot and mesocosm experiments).

We developed a list of inclusion/exclusion criteria to guide and standardize the literature screening process (Additional file 3).

#### Study validity assessment

We focused on external validity, i.e., the relevance of each publication regarding our study question. To achieve this, we systematically extracted study characteristics that give a reliable overview of relevance and validity. These characteristics encompassed the geographical range, number of sampling sites, duration of the study, type of design employed, statistical analyses conducted, the description of the ISG monitoring method, and the ISG and AMP focus (see data coding strategy beneath).

### Data coding and mapping method

The data extracted from the articles included bibliographic information, study design and field type, and details on ISGs and AMPs studied. The data coding strategy consists in the combination of five tables linked by a unique article identifier (codes and conditions detailed in Additional file 4). The full database resulting from this process is available in Additional file 5. The first table, “Article References”, contains the articles bibliographic information. The second table, “Study Characteristics”, contains information about study location and design. The third table, “Crop Type”, contains information about the investigated field types and detailed crops. The fourth table, “AMP”, contains information about the studied AMPs categories and details. Here we state if the AMP is studied in comparison with a control with no intervention (before-after tillage or tillage versus no tillage), or if the focus is management options of the AMP (e.g. tillage depth, frequency or machinery), as showed in left part of Fig. 5. The fifth table, “ISG” contains information about the studied ISGs diversity measures and monitoring methods used (see global database and individual tables in Additional file 5).

To assess the repeatability of the data coding process, we compared the data extracted by the two different reviewers (CT and MP) on a subset of 50 articles. For each data type to be extracted, we assessed the reviewer’s agreement as the percentage of fit. Cases of disagreement were discussed to improve the collection repeatability (i.e., by clarifying the definition of a variable, reformulating the different categories of a variable in the case of multiple choices, or adding additional variables if necessary).

The mapping of the relevant evidence was described with figures and tables. Combination of AMPs, ISGs and methods were tabulated in heatmaps to illustrate the volume of evidence and to identify knowledge clusters and gaps.

## Review findings

### Literature searches and screening

We gathered 20 150 references after removal of duplicates using WOS and CABI, and 12 additional references with Google Scholar. At the end of the screening process, we obtained 1 208 publications and 22 documents from grey literature that were included in this systematic map (Fig. 1).

### Mapping of the relevant evidence

#### Studied areas and chronological evolution

**Fig. 2:**
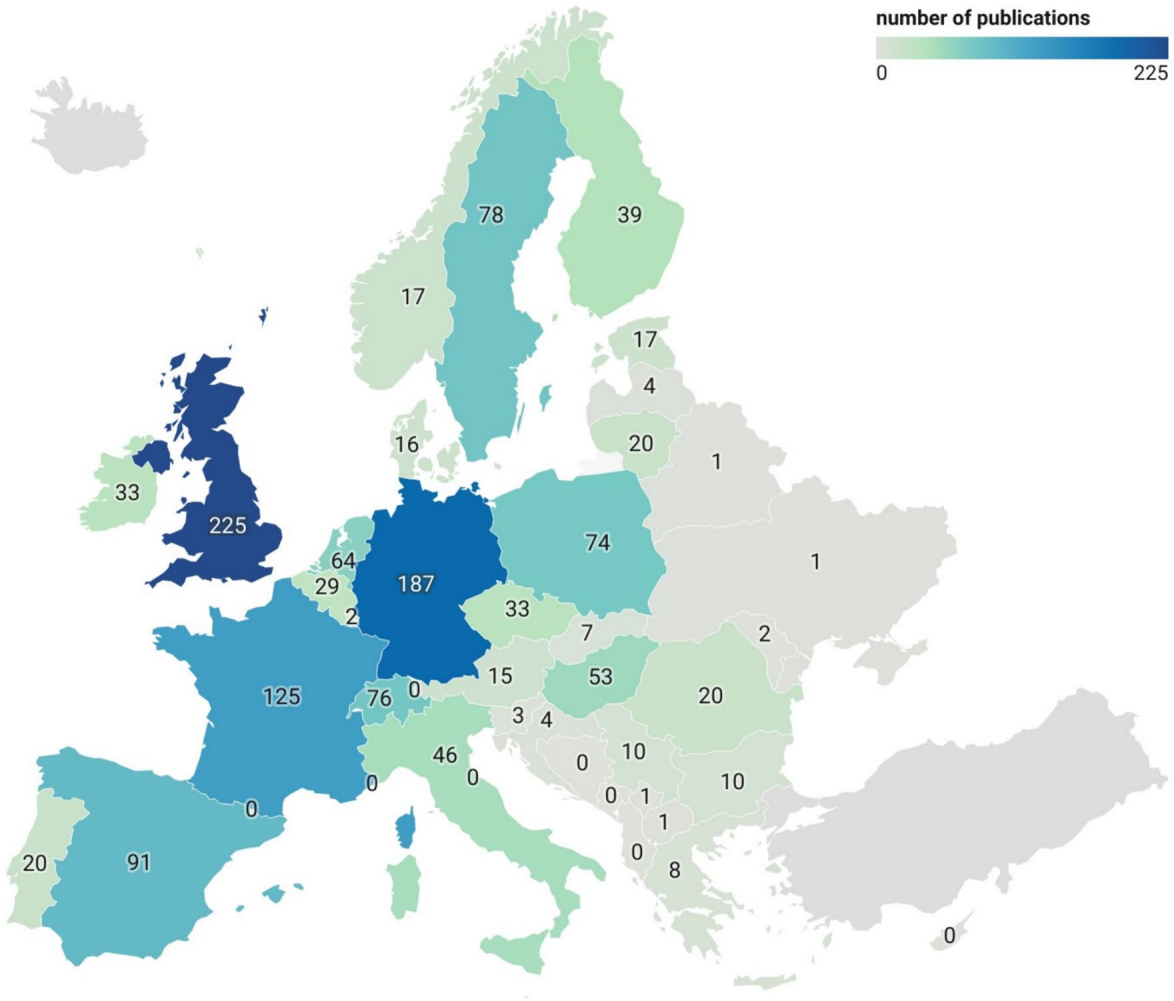
Map of the European distribution of the studies included in the systematic map. Iceland and Turkey (in grey) were not included in the search

The studies included in this map were mainly conducted in the United Kingdom (UK, 17% of the studies), Germany (14%), France (9.3%), Spain (6.8%), Sweden (5.8%), Switzerland (5.7%) and Poland (5.5%) that together represent 64% of the studies (Fig. 2).

**Fig. 3:**
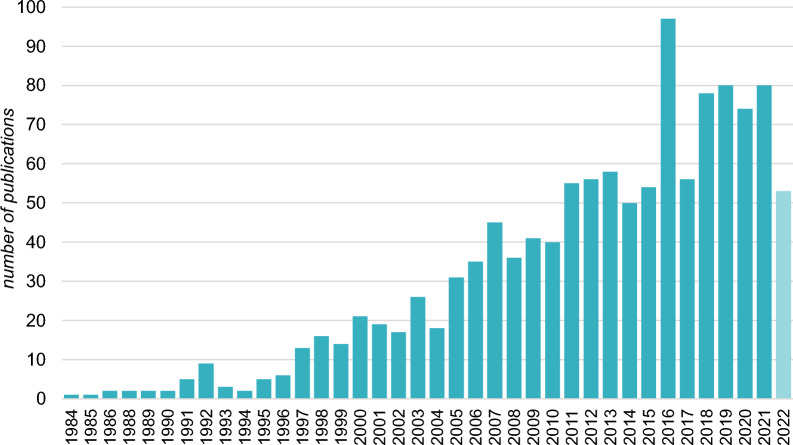
Number of studies included in the systematic map from each year of the study period

The included studies were published from 1984 to April 2022 as illustrated in Fig. 3. The number of published studies per year massively increased over the last 30 years, aligning with the general trend observed in the broader field of ecology and environmental sciences [36]. The most prolific years were 2016, and from 2018 to 2021. References from 2022 were included up to April 19th as the extraction of references was completed on that date.

#### Field types, comparators and experimental designs

**Fig. 4:**
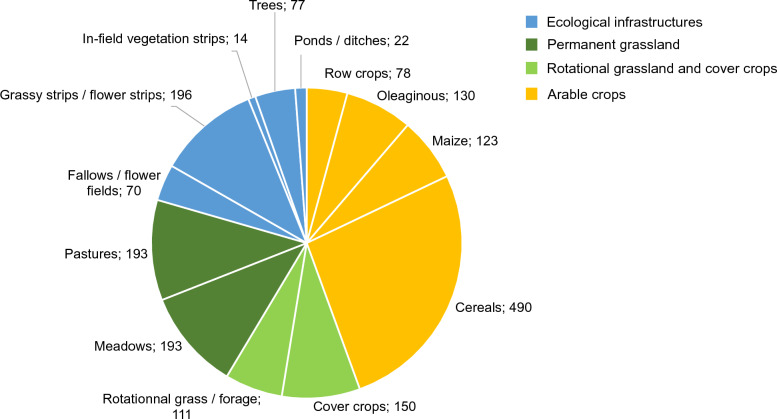
Number of publications for each field type of interest

The included studies were conducted in arable crops, temporary and permanent grasslands, and ecological infrastructures. A quarter of the studies (307) included more than one crop type: 56% of the publication focused on arable crops, 46% on grasslands, and 27% on ecological infrastructures. The first study about ecological infrastructures was published in 1994. The detailed crop types recorded in the systematic map are given in Fig. 4.

The majority of studies (90%) compared the intervention with a control, while 7% compared the state of the population before and after the intervention. Both comparators were often combined in Before-After Control-Impact (BACI) designs. There are 15.7% of the studies comparing two or more interventions.

On-farm design represented 51.9% of the studies (i.e., comparing treatments on different sites or plots but not randomized or replicated), experimental design 41.6% (field trials with randomized spatial replication, mainly split-plot designs with replication on small plots at 1 site), and monitoring studies 5.8% (count of individuals in defined areas, searches done by hunt/observation).

Regarding the temporal scale of the studies, 32.8% of the publications studied the long-term effects of AMPs (more than 6 years), while 3.7% their immediate or short-term effects (from 1 day to a few months, but less than the growing season). Most studies assessed the impacts of AMPs in an intermediate scale, i.e., at the scale of the growing season (29%) or for 2 to 5 years (25.5%). Overall, 10% of the publications studied the effects of AMPs at several temporal scales. The choice of the temporal scale is driven by the type of AMP studied. For example, pesticide use was often studied for its impact on a short-term, while organic farming or EI implementation were investigated on a longer term. Long-term effects were assessed with on-farm designs twice as much as with experimental designs. Most studies focus on the response of ISGs at the field scale (83%) and 19.4% of the studies took the landscape scale into account. As expected, experimental designs were used quite exclusively for assessing field-scale effects, with 4 studies taking into account the landscape scale. Impacts of AMPs on biodiversity at the farm scale were assessed in 89 studies (7.4%), either by monitoring all the fields of the farm or a representative subset. The spatial scale of the studies is linked to the mobility of ISGs, for example birds are quite exclusively studied at the landscape scale while annelids are studied at the field scale or even the sampling plot scale.

Ecosystem services were reported in 151 publications (12.5%), with a predominant focus on yield, which appeared in 98 publications (8.1%). Other ecosystem services such as regulation services, including pest regulation (26 publications, 2.2%) and pollination (14 publications, 1.2%), were frequently addressed. The costs associated with ISG monitoring were never mentioned.

Finally, 44 studies did not conduct inferential statistics (descriptive results only).

#### Intervention: agricultural management practices (AMPs)

**Fig. 5:**
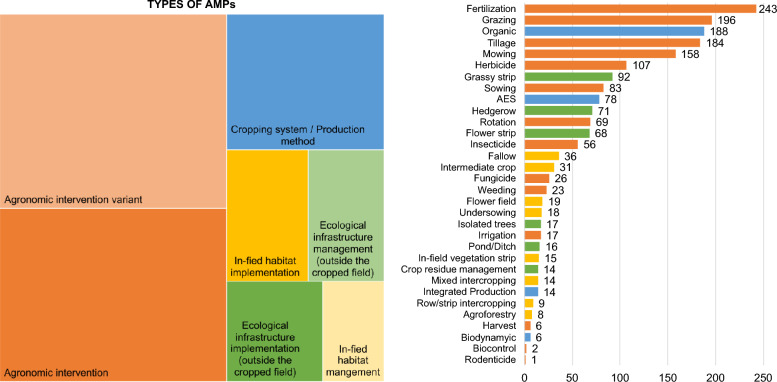
Allocation of AMP types combining individual AMPs (left), and number of publications for individual AMPs (right). Agronomic interventions are in orange, In-field habitats in yellow, Ecological infrastructures in green, and production methods in blue

As shown in Fig. 5, the most studied AMPs are fertilization (in 22.2% of the publications) followed by grazing, organic farming, tillage, and mowing (in more than 10% of the publications each). We divided AMPs in three main groups, each divided in two sub-groups for intervention and management:

- Most studied AMPs are agronomic interventions (e.g., tillage, fertilization, pesticide application, mowing) and their management options (e.g., method, dose, frequency), in orange in Fig. 5
- The second group of studied AMPs is in-field habitat implementation (e.g., implementation of cover crops with intermediate crops, undersowing or intercropping, implementation of fallows, flower fields and agroforestry) and their management options (e.g., seed mixture, sowing density), in yellow in Fig. 5
- The third group of AMPs is ecological infrastructure implementation or presence (e.g., grass and flower strips, hedgerows, ponds) and their management options (e.g., mowing regime, trimming, seed mixture), in green in Fig. 5.

We additionally created a fourth group for AMPs concerning the whole cropping system, encompassing various practices without studying them individually, e.g., organic farming, integrated production, soil conservation agriculture (in blue, Fig. 5). It also includes studies that compared areas with and without AES adoption, without defining or separating the practices included in the schemes.

Among AMPs recorded in this map, 153 articles (12.7%) mentioned that the studied AMP was part of an AES, meaning that the farmer received subsidies for its implementation.

Some studies aimed at assessing edge or spillover effects. To do so, 64 publications (5.3%) studied the effects of the AMP on the adjacent crop or habitat. These studies assessed the effects of an EI on the adjacent cropped field, or the effect of organic farming methods on the adjacent semi-natural habitats. A total of 137 publications (11.4%) studied the effect of the distance to the field margin or habitat margin, either by sampling at different distances from the margin or by comparing the edge (first rows of the crop) and the crop interior.

#### Population: indicator species groups (ISGs)

**Fig. 6:**
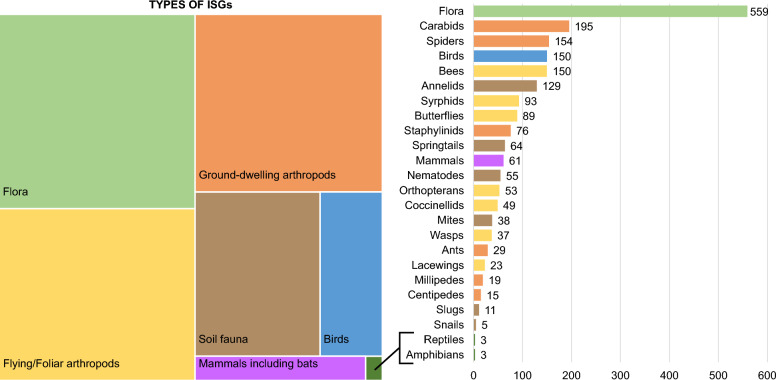
Allocation of ISGs combining individual ISGs (left), and number of publications for individual ISGs (right). Flora is in light green, foliar arthropods in yellow, soil fauna in brown, ground-dwelling arthropods in orange, birds in blue, mammals in purple and reptiles with amphibians in dark green

**Fig. 7:**
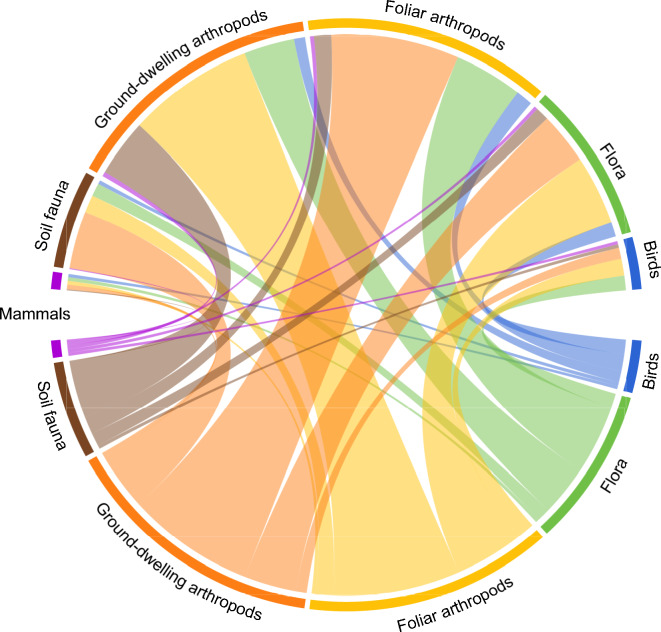
Chord diagram of ISGs studied by pair. The thickness of the connections represents the number of studies combining two ISGs. Amphibians and reptiles do not appear as they were never combined with another ISG

The most studied ISG is flora, appearing in 46.4% of the publications (Fig. 6), often together with one or multiple other ISGs (Fig. 7). Regarding animals, the main ISGs (each appearing in more than 10% of the publications) are carabids, spiders, birds, bees, and annelids. In 89% of the publications, the ISGs were identified at the species level, and 18.7% studied a subgroup of the ISG, for example a specific species, genus, family or functional group within the ISG (e.g., brown hare, bumblebees, wolf spiders, granivorous carabids).

Looking at the number of studies published each year for individual ISGs, there was no outstanding temporal variation observed but within the most studied ISGs mentioned above, bees and birds began to receive more attention slightly later, around the year 2000. From the 2000s, the diversity of ISGs studied every year increased, which is probably due to the exponential increase in publications.

Main combinations between ISGs are the followings: ground-dwelling arthropods with foliar arthropods, soil fauna is often monitored together with ground-dwelling arthropods, and flora is also often assessed when ground-dwelling and foliar arthropods are, as illustrated in Fig. 7. There was no combination of reptiles and amphibians with other ISGs.

**Fig. 8:**
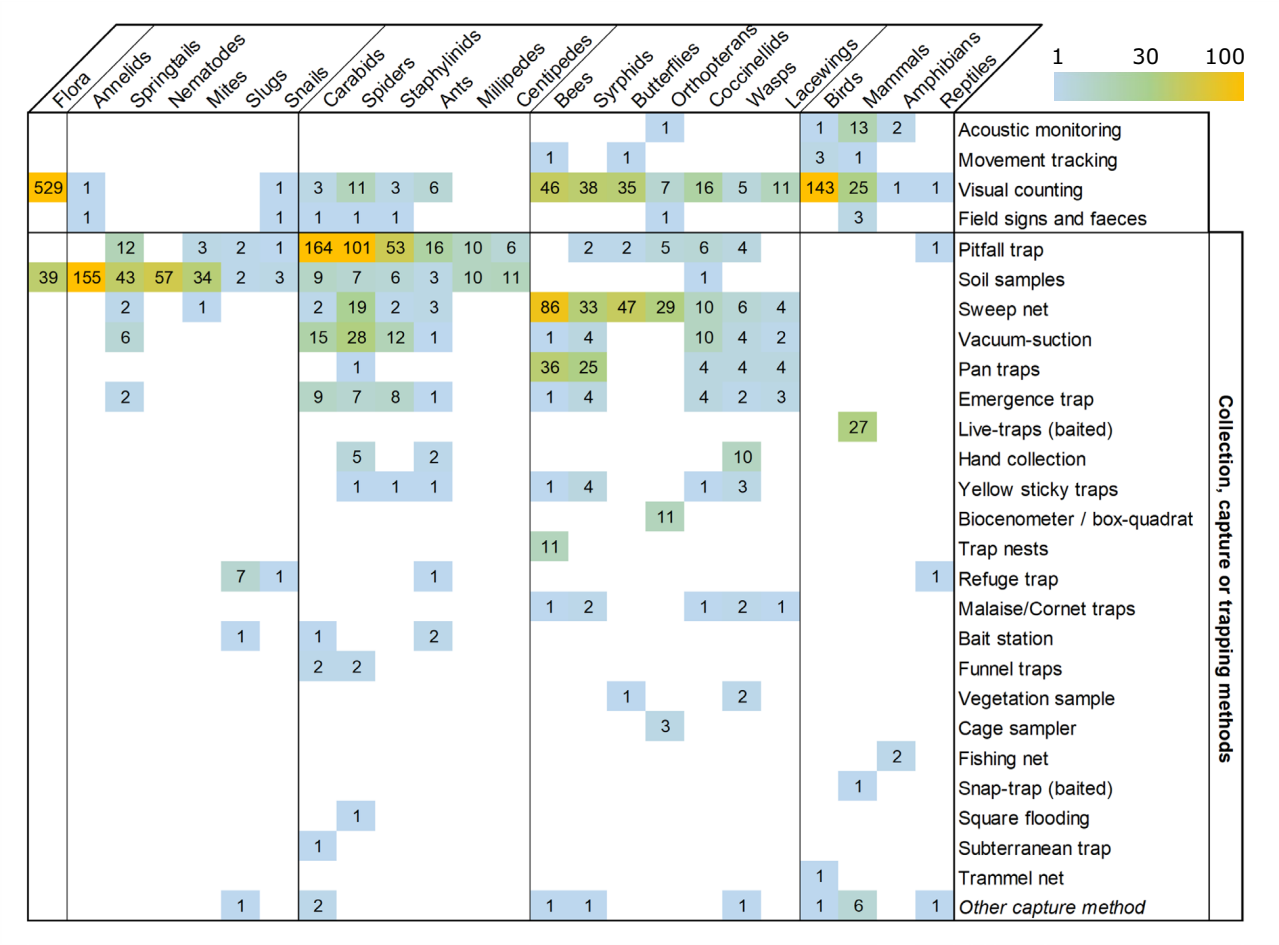
Heatmap of the monitoring methods used (horizontal) for studying the ISGs (vertical). Numbers refer to the number of publications. ISGs are grouped according to Fig. 6 i e. from left to right: flora, soil fauna, ground-dwelling arthropods, flying and foliar arthropods, and others (birds, mammals, amphibians and reptiles). Color scale from 1 to 100 with 100 including all values beyond 100

Monitoring methods recorded for each ISG are shown in Fig. 8. Some biodiversity monitoring methods are widely used for recording multiple ISGs of the same group: soil samples followed by various extraction methods for soil fauna, pitfall traps for ground-dwelling arthropods, sweep nets for foliar and flying arthropods. Visual counting encompasses point-count and territory mapping for birds, quadrats and transects for arthropods and flora. Flora was either monitored by weed seed bank sampling or visual counting of individuals and species. This visual counting was generally done in the field, sometimes samples were taken to the lab but this distinction was not included in the mapping. Detailed monitoring methods are reported in the database (Appendix 5).

Only nine publications used DNA extraction for the identification of samples of annelids, bees, nematodes and slugs. There was no visible trend towards a change in monitoring methods used for most ISGs, besides the apparition of new technologies as DNA extraction, GPS tracking and acoustic monitoring, they are still marginally used (Fig. 8). For mammals after the year 2005, these technologies together with visual counting seemed to be preferred to capture methods (mainly live traps).

#### Outcomes: diversity measures

**Fig. 9:**
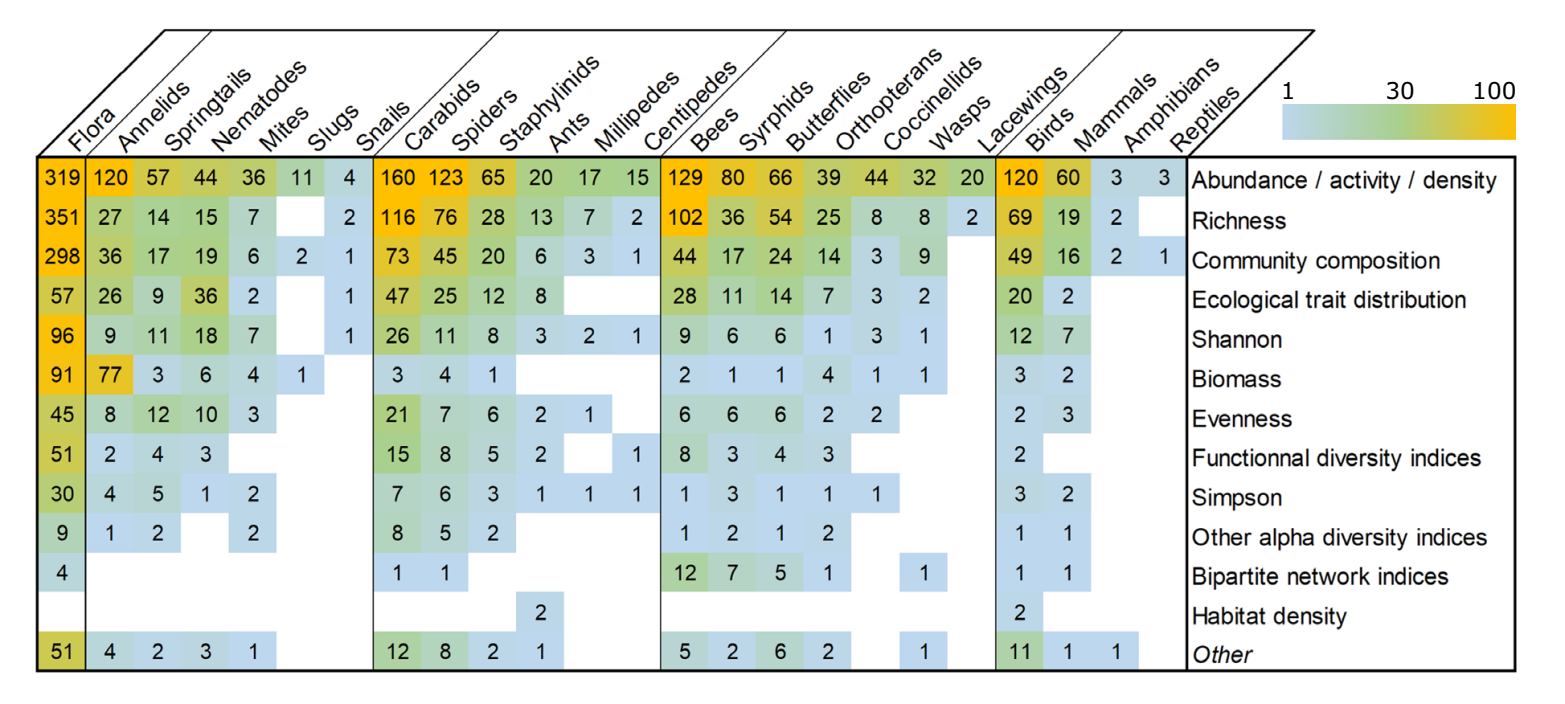
Heatmap of the diversity measures (outcomes, vertical) used for studying the ISGs (horizontal). Numbers refer to the numbers of publications. ISGs are grouped according to Fig. 6 i e., from left to right: flora, soil fauna, ground-dwelling arthropods, flying and foliar arthropods, and others (birds, mammals, amphibians and reptiles). Color scale from 1 to 100 with 100 including all values beyond 100

The main measured outcome is abundance, which was reported as abundance, activity or density, followed by species richness, evenness and Shannon index of diversity (Fig. 9). Even if they were not targeted in the search strings, many studies also reported the effects of AMPs on community composition (often analyzed with ordination methods, PERMANOVA or dissimilarity indices), or on the distribution of ecological traits in the community (e.g. size, diet, dispersal ability).

A total of 35% of the ISG monitoring were reported with abundance and/or biomass only. In this context, in 65% of cases, the abundance/biomass reported is that of the whole group without further identification (e.g., the taxonomic level used is the family Carabidae for carabids, the superfamily Apoidea for bees, and the class Aves for Birds). The percentage varies a lot between ISGs: 19% and 27% respectively for birds and mammals which are large groups often studied with a specific focus on one of few species of interest, more than 70% for the soil fauna, and more than 65% for all arthropods.

#### Associations between AMPs and ISGs, identification of hotspots

**Fig. 10:**
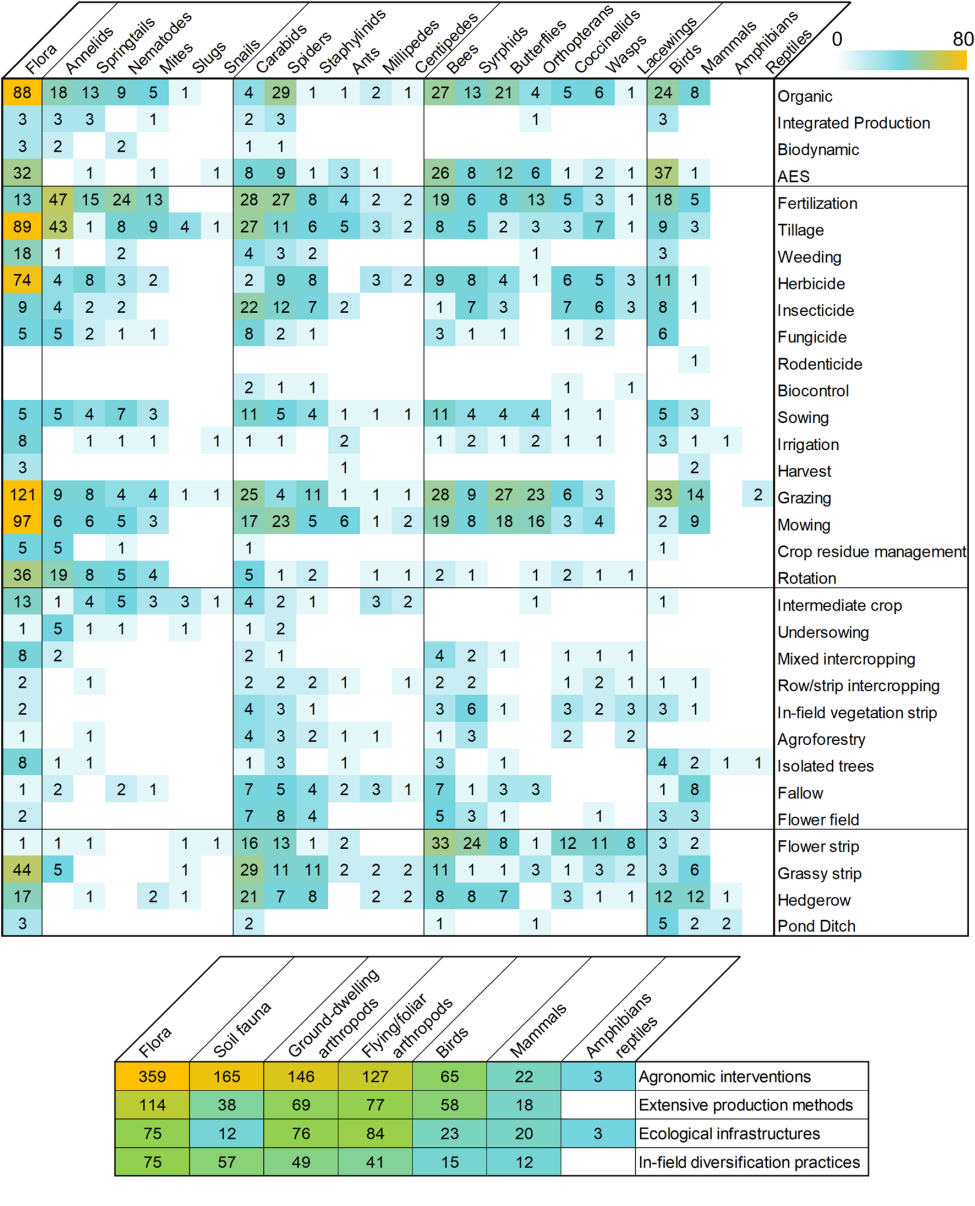
Heatmap of the number of studies assessing effects of AMPs on ISGs. Numbers refer to the number of publications (one publication can report the effect of several AMPs on several ISGs). ISGs are grouped according to Fig. 6 i e., from left to right: flora, soil fauna, ground-dwelling arthropods, flying and foliar arthropods, and others (birds, mammals, amphibians and reptiles). AMPs are grouped according to Fig. 5 i e., production methods, agronomic interventions, in-field habitat implementation, ecological infrastructures. *AES* Agri-Environmental Scheme. Color scale from 1 to 80 with 80 including all values beyond 80. Below, the small heatmap is a summary of the findings for more clarity

The effect of non-conventional production methods and AES application has been assessed on most of the ISG groups, mostly on flora, spiders, bees, butterflies and birds (Fig. 10). However, the effects of specific ecological infrastructures implementation and management were mainly assessed on ground-dwelling and flying arthropods, particularly natural enemies and pollinators. A similar trend is observed for agroecological practices aimed at increasing habitat and resources within fields, such as intercropping. There was limited evidence of side effects of rodenticides (with one study on non-target mammals) and biocontrol (two studies on generalist natural enemies, see Fig. 10).

From the ISG perspective, carabids, spiders and staphylinids are commonly used as indicators for assessing the influence of a wide range of AMPs. Bees and syrphids are also studied in relation to many AMPs, particularly flower strips (Fig. 10). Butterflies and orthopterans are often used as indicators of grassland mowing and grazing impacts on biodiversity, along with flora. Flora, as the main ISG recorded, was particularly used for assessing the effects of tillage, herbicides, organic farming, mowing and grazing. Soil fauna was mostly studied regarding agronomic interventions, mainly the reaction of annelids to fertilization, tillage and rotation, and the influence of fertilization practices on nematodes, springtails and mites (mites and springtails are often studied together). Finally, birds are used as indicators of the influence of AMPs at the landscape scale, and mainly for organic farming, AES adoption, grazing, ecological infrastructures, and the indirect impacts of fertilization, tillage and pesticides.

### Limitations of the map

#### Limitations due to the search strategy

Several risks of bias have emerged due to the choices we made in the search. First, we limited our search to English language, leading to risk language bias, i.e. missing literature written in other languages. Then, we searched grey literature in English and French on the main Swiss specialist websites, which encompass a large number of documents. However, few of them fitted our inclusion criteria, mostly because most grey literature in the area of agronomy and biodiversity is simplified scientific communication of general knowledge on a topic, but not documents reporting tested effects of agricultural practices on biodiversity. Limiting our research to Swiss websites risks publication bias regarding grey literature. Finally, even if we chose to include a large range of ISGs representing diverse trophic levels and scales of indication (habitat, field or landscape) based on previous research [19], we chose to exclude some taxonomic groups (e.g. Opiliones) which risks a type of selection bias.

#### Semantics and interpretation

While coding, we grouped AMPs in the different categories with care, aiming to preserve as much details as possible. However, AMPs grouping could have been done slightly differently by referees with differing background knowledge or experience, especially when the information was hard to find, or even contradictory within the text of a publication. Moreover, the same practice or technique can have different names depending on the time period, country, or context, which leads to some interpretation from the reader, and does not facilitate the coding. This shows the importance of semantics in science, and to define clearly the terms used in each publication.

#### Lowland vs mountain

Possibly, studies relating to investigations located in upland or mountain areas have been inadvertently included if the authors did not explicitly mention the geographical context or because ambiguity arises due to lack of universal definition of lowland. Indeed, the classification of geographical areas as lowlands, uplands or mountains can vary between countries and is often not clearly stated in the literature. Elevation, slope and accessibility are part of the criteria used to qualify an area as lowland or not. For example, the Swiss plateau average height is between 400 and 700 m above the sea level (a.s.l.), while British upland is usually defined as land 300 m a.s.l. We were not able to use a universal maximal elevation as a criterion, we thus chose to refer to the author's definition of their study areas to exclude articles in uplands or mountains when a doubt persisted. Nevertheless, we provide in Additional file 7 the list of publications that were excluded only because they are in uplands, mountainous or alpine areas (we extracted information as for the included articles for the purpose of a following synthesis).

#### Ecological infrastructures could be underrepresented

The terms dedicated to AMPs in the search string did not include the individual EIs presented in the review findings. We rather included terms defining the way EIs are implemented or qualified in the literature: “ecological compensation”, “biodiversity promotion” and “ecological focus” areas, “agri-environment schemes” or “AES”, or “semi-natural habitats”. When evaluating the comprehensiveness of the literature search with a test list of 90 articles, it appeared that the research string had a very good result and represented efficiently the EI implementation practices. We are thus confident in the search string and the results obtained. However, we should acknowledge that some relevant publications about grassy strips, wild-flower strips, hedgerows, and pond management may be missing if their title, abstract and keywords did not mention any of the search terms used for AMPs and their management options (see Additional file 3).

## Conclusions

### Knowledge clusters and gaps

#### Knowledge clusters

- Flora response is widely studied for all kind of farming practices, as well as carabids, spiders, and bees.
- Organic farming, fertilization, tillage, grazing and mowing are well documented.
- Annelids is the most studied indicator species group in regard to agronomic interventions that impact soil structure (e.g., tillage, fertilization, crop rotation, crop residue management).
- Bees and syrphids are the most used species groups to assess flower strips impacts, while carabids are preferred for assessing grassy strips and hedgerows.
- Butterflies and orthopterans were mostly investigated in grassland for assessing mowing and grazing effects.
- Birds is the most used indicator species group to assess agri-environmental schemes efficiency, at the landscape scale.

#### Knowledge gaps

- The review findings are not representative of all Europe as some countries are largely underrepresented (e.g., Latvia, Croatia, Slovenia, Moldova, Ukraine) or not represented at all (e.g., Albania, Bosnia, Herzegovina), thus care should be taken before drawing general conclusions.
- Amphibians, reptiles, snails, slugs, millipedes and centipedes are poorly documented.
- Field evidence of the impact of harvest, rodenticides and biocontrol is very scarce.
- Field evidence of the impact of insecticides is surprisingly low.
- Diversification practices such as intercropping, undersowing, intermediate cropping, and agroforestry are less studied than the other farming practices groups. This gap is even bigger regarding soil fauna. This result is surprising regarding intermediate cropping, because winter cover crops are widely adopted in Europe and known for their multifunctionality (e.g. weeds regulation, soil fertility, water protection, carbon sequestration…).
- Ecological infrastructures effects are well documented except for soil fauna.
- Besides agri-environmental schemes, few farming practices are assessed at the landscape scale.

The topic of analyzing the impact of agricultural practices on biodiversity has been the subject of recent synthesis work, listing and reporting meta-analyses [41–43] at the global scale. Their findings also show that tillage and fertilization are the most individually studied practices, however they converge saying that the combination of practices (including AES, organic farming, and combinations of biodiversity promotion practices) are more representative of the real fields conditions, and are more likely to show concordant effects on several biodiversity groups [42–44].

### Implication for research

We encourage researchers to use the database provided by this systematic map as a tool for gathering article references on their topic of interest for literature syntheses, and potential future systematic reviews (meta-analyses), particularly on the knowledge clusters identified above. To that end, this systematic map should be updated each 3 to 5 years, following the CEE recommendations.

The systematic map highlights that flora is over-represented compared to other indicator species groups; especially reptiles, amphibians and snails are rarely assessed. Indeed, Flora is more studied (i) in link with the search for methods for weed control and (ii) in relation to other biodiversity groups as herbivores, pollinators. However, comprehensive, empirical assessment of biodiversity (indicators) within the diverse bio-physical compartments of the agricultural landscape is rare or even absent. The over-representation of some groups (i.e., looking always at the same indicators) leads to lacking information on other groups that could have interesting different, possible antagoniassociated ecological processesstic, responses. Future research should focus on the impact of agricultural management on biodiversity groups that are, until now, poorly assessed, because contradictory effects could lead to different policy measures. Moreover, having more complete data is essential to know if some groups can be surrogates for other groups. In general, future research should focus on the knowledge gaps presented above.

Assessments of edge and spillover effects or distance decay from e.g., ecological infrastructure into fields are less common than we thought (e.g. beneficial species and their associated ecosystem services—pollination, pest regulation-, weeds and slugs). More research should be conducted to enlighten the potential of spillover of the different indicator species groups, especially on soil fauna and ground-dwelling arthropods, and assess the link between ISGs diversity measures and ecosystem service delivery in the cropped fields.

We also showed that methods used in the agroecological research field did not change drastically since the 80 s. Emergent technologies are still nowadays rarely used. Chosen monitoring methods are either visual counting (requiring experts’ knowledge) or invasive (destructive sampling with traps or captures). These methods require heavy field campaigns and determination work that could be relieved thanks to new technologies. For instance, DNA identification is not widely used for the indicator species groups selected for the map in comparison to micro-organisms [38], but its democratization would potentially increase the level of precision in research results. Indeed, time and money restraints often lead research projects to skip further taxonomic identifications and use only the general abundance of a taxonomic group [37]. Metrics reported were dominated by abundance at high taxonomic level (e.g., family), an important measure for biodiversity assessment but not satisfactory for understanding how communities react to disturbances and the associated ecological processes (similar findings in [39]). Functional diversity indices are rarely used, and the distribution of ecological traits of indicator species groups rarely investigated, we would recommend increasing their use in future research. Indeed, the response of ecological traits of indicator species groups, regardless of their taxonomic or phylogenetic proximity, can help identify and understand those processes.

Moreover, the map showed a minority of studies at the farm scale. Studying biodiversity at the farm scale is a challenge for research because the more the fields of a farm are dispersed in the landscape, the more it is influenced by neighboring farmers' practices. No included study took the cohesion/dispersion of the farm field in the landscape into account. The main approach was to sample all fields or a subset of the farm fields. Being able to find biodiversity indicators that are reliable at the farm scale is necessary to provide tools for farmers to evaluate the impact of their management practices on the indicator species groups and the environment. This is the objective of the Indicate research program.

Finally, the process of full text screening and data coding highlighted deficiencies in the way research studies are reported (e.g., missing years, location, crop type or monitoring methods in the article, no species list when applicable). In order to improve the quality of the publications and replicability of the studies, the scientific community and journals should work on common standards for publications, for example by providing a checklist of minimal information that should be provided. Also, as mentioned before, a work on semantics by defining technical terms (e.g., practices and methods) would greatly facilitate the synthesis of knowledge.

### Implication for policy or management

The database provided by this systematic map references reliable methods and indicators for assessing biodiversity in the agricultural context. This map will therefore be useful for stakeholders that want to set up a monitoring, especially for optimizing the combination of methods to assess a wide range of indicator species groups known to respond to the assessed practices. This map is also a tool for identifying research groups working on specific subtopics, and expert taxonomists for the different indicator species groups, facilitating collaborations with scientists and the knowledge transfer.

As mentioned, the knowledge available on the subject is condensed on limited indicator species groups. We can question the choice of biodiversity groups studied, which is usually driven by public interest and research fundings, but also human habits and previous knowledge (i.e., researchers tend to make research based on previous findings to go further). Exploratory research into forgotten biodiversity groups should be encouraged, because understanding the response of poorly studied biodiversity groups is necessary to make some recommendation for management, especially for very different organisms whose response is different from the surrogate indicator usually chosen [40]. Studying the correlation between groups responses is the next important step to define the most representative indicator(s) for biodiversity.

Last but not least, such a systematic map provides the foundation of further information extraction, i.e., the effects of farming practices on multiple indicator species groups from selected studies to be transmitted to extension services and farmers for implementation.

### Supplementary Information

Additional file 1. ROSES checklist for systematic map reports

Additional file 2. Search strings and test list

Additional file 3. Inclusion/exclusion criteria

Additional file 4. Data coding—codes and conditions

Additional file 5. Systematic map database

Additional file 6. List of excluded articles at full text screening

Additional file 7. List of mountain articles

## Data Availability

The datasets supporting the conclusions of this article are included within the article (and its additional files).

## Declarations

### Declarations

**Ethics approval and consent to participate**

Not applicable.
