# Supplementary material for: What evidence exists on the effect of the main European lowland crop and grassland management practices on biodiversity indicator species groups? a systematic map
Source: Environ Evid. 2024 Aug 17;13:20. doi: 10.1186/s13750-024-00347-0 (PMC11329403; doi:10.1186/s13750-024-00347-0)
Supplement: Supplementary file 1 — Additional file 1. ROSES checklist for systematic map reports [file 13750_2024_347_MOESM1_ESM.pdf]

Item number	Section/sub-section	Topic	Description	Further explanation	Checklist/meta-data	Author response
1	Title	Title	The title must indicate that it is a systematic map, and should indicate if it is an update/amendment: e.g. "…A systematic map update."	The title should normally be the same or very similar to the review question.	Meta-data	What evidence exists on the effect of the main European lowland crop and grassland management practices on biodiversity indicator species groups? A systematic map
2	Type of review	Type of review	Select one of the following types of review: systematic map, systematic map update, systematic map amendment	See CEE Guidance on systematic mapping [1], and on amendments and updates [2]	Meta-data	systematic map
3	Authors' contacts	Authors' contacts	The full names, institutional addresses and email addresses for all authors must be provided.		Checklist	Yes
4	Abstract	Structured summary	The abstract of the manuscript must not exceed 500 words and must be structured into separate sections: Background, the context and purpose of the review, including the review question; Methods, how the review was performed (specifically mention search strategy, inclusion criteria, critical appraisal (optional), meta-data extraction and coding, and narrative synthesis); Results, the main findings, including results of search and assessment of evidence base; Conclusions, brief summary and potential implications for policy/management and research.		Checklist	Yes
5	Background	Background	Describe the rationale for the review in the context of what is already known. Reviews must indicate why this study was necessary and what it aims to contribute to the field.	A theory of change and/or conceptual model should be presented that links the intervention or exposure to the outcome.	Checklist	Yes
6	Stakeholder engagement	Stakeholder engagement	The actual role of stakeholders throughout the review process (e.g. in the formulation of the question) must be described and explained (using a broad definition of 'stakeholder', including e.g. researchers, funders and other decision-makers; see [3])		Checklist	Yes
7	Objective of the review	Objective	Describe the primary question and secondary questions (when applicable).	The primary question is the main question of the review. The secondary questions are usually linked to sources of heterogeneity (effect modifiers).	Checklist	Yes
8		Definition of the question components	Provide reference to the question key elements, e.g. population(s), intervention(s)/exposure(s), comparator(s), and outcome(s).	For other question types see [4,5]	Meta-data	Population (P): The biodiversity indicator species groups (ISGs)<br>Intervention (I): The European lowland agricultural management practices (AMPs)<br>Comparator (C): The comparison before/after AMP interventions, between AMPs and controls, or between different AMPs<br>Outcome (O): Measure of change of the ISGs (i.e., diversity, abundance, or evenness)
9	Methods	Protocol	Provide citation, DOI or open-access link to published protocol.	The protocol should be peer-reviewed and publicly available online (open access).	Meta-data	https://doi.org/10.1186/s13750-022-00280-0
10		Deviations from protocol	Describe any ways in which the final methods of the review deviate from those set out in the protocol along with a justification.		Checklist	Yes
11	Searches	Search strategy	Detail the search strategy used, including: database names accessed, dates of searching, institutional subscriptions (or date ranges subscribed for each database), search options (e.g. 'topic words' or 'full text' search facility), efforts to source grey literature, other sources of evidence (e.g. hand searching, calls for evidence/submission of evidence by stakeholders).		Checklist	Yes

12		Search string	Provide Boolean-style full search string and state the platform for which the string is formatted (e.g. Web of Science format)		Meta-data	Formated for Web Of Science<br>TI/AB/AK=(ISG keywords)<br>AND<br>TI/AB/AK=(((ISG keywords) NEAR/3 (richness OR composition$ OR abundan* OR diversity OR evenness OR number$ OR assemblage$ OR communit* OR population$)) OR (species NEAR/3 (richness OR composition$ OR abundan* OR diversity OR evenness OR number$ OR assemblage$ OR communit* OR population$)) OR shannon OR simpson)<br>AND<br>TI/AB/AK=("soil preparation" OR till* OR plough* OR fertili* OR amendment* OR compost* OR biochar* OR manur* OR sow* OR planting OR irrigat* OR watering OR "crop protection" OR "pest control" OR "pest management" OR "weed control" OR pesticide$ OR insecticide$ OR herbicide$ OR rodenticide$ OR bactericide$ OR harvest* OR reaping OR "residue management" OR "crop residue" OR mow* OR cutting OR hay OR silage OR grazing OR pasture$ OR husbandry OR livestock$ OR cattle$ OR "cover crop" OR "catch crop" OR "intermediate crop" OR "high nature value" OR hnv$ OR "agri-environment schemes" OR aes OR "semi-natural" OR snh$ OR "ecological compensation" OR eca$ OR "biodiversity promotion" OR bpa$ OR "ecological focus" OR efa$ OR land$use OR organic OR conventional OR agro$ecology OR agro$forestry OR "crop rotation")<br>AND<br>TI/AB/AK=(farm* OR agri* OR crop* OR grassland* OR arable OR cultivated)<br>AND<br>ALL=(Albania OR Andorra OR Austria OR Belarus OR Belgium OR Bosnia OR Herzegovina OR Bulgaria OR Croatia OR Cyprus OR Czech* OR Denmark OR Estonia OR Finland OR France OR Germany OR Greece OR Hungary OR Ireland OR Italy OR Kosovo OR Latvia OR Liechtenstein OR Lithuania OR Luxembourg OR Moldova OR Monaco OR Montenegro OR Netherlands OR Macedonia OR Norway OR Poland OR Portugal OR Romania OR “San Marino” OR Serbia OR Slovakia OR Slovenia OR Spain OR Sweden OR Switzerland OR Ukraine OR “United Kingdom” OR “UK” OR England OR Britain OR Scotland OR Wales OR Europe*)
13		Languages - bibliographic databases	List languages used in bibliographic database searches		Meta-data	English
14		Languages – grey literature	List languages used in organisational website searches and web-based search engines		Meta-data	English, French
15		Bibliographic databases	Provide the number of bibliographic databases searched		Meta-data	2
16		Web-based search engines	Provide the number of web-based search engines searched		Meta-data	1
17		Organisational websites	Provide the number of organisational websites searched		Meta-data	3
18		Estimating comprehensiveness of the search	Describe the process by which the comprehensiveness of the search strategy was assessed (i.e. list of benchmark articles)		Checklist	Yes
19		Search update	Describe any update to searches undertaken during the conduct of the review	Compulsory (if update performed). A search update is good practice if original searches were performed more than two years prior to review completion.	Checklist	Yes
20	Article screening and study inclusion criteria	Screening strategy	Describe the methodology for screening articles/studies for relevance. Methods for consistency of screening decisions (at title, abstract, and full texts levels) checking must be described.		Checklist	Yes
21		Inclusion criteria	Describe the inclusion criteria used to assess relevance of identified articles/studies. These must be broken down into the question key elements (e.g. relevant subject(s), intervention(s)/exposure(s), comparator(s), outcome(s), study design(s)) and any other restrictions (e.g. date ranges or languages).		Checklist	Yes

22	Critical appraisal	Critical appraisal strategy	Describe here the method used for critical appraisal of study validity (including assessment of individual studies and the evidence base as a whole). Describe how repeatability of critical appraisal of study validity was tested.	Optional	Checklist	n/a	
23		Critical appraisal used in synthesis	Describe how the information from critical appraisal was used in synthesis.	Compulsory if critical appraisal performed	Checklist	n/a	
24	Meta-data extraction and coding strategy	Meta-data extraction and coding strategy	Describe the method for meta-data extraction and coding for studies, providing lists of variables that will be extracted as meta-data and those that will be coded. Describe how repeatability of meta-data/data extraction and coding was tested.		Checklist	Yes	
25		Approaches to missing data	Describe any process for obtaining and confirming missing or unclear information or data from authors.		Checklist	Yes	
26	Data synthesis and presentation	Narrative synthesis strategy	Describe methods used for narratively synthesising the evidence base in the form of descriptive statistics, tables (including SM database) and figures.		Checklist	Yes	
27		Knowledge gap and cluster identification strategy	Describe the methods used to identify and/or prioritise key knowledge gaps (unrepresented or underrepresented subtopics that warrant further primary research) and knowledge clusters (well-represented subtopics that are amenable to full synthesis via systematic review).		Checklist	Yes	
28		Demonstrating procedural independence	Describe the role of systematic reviewers (who have also authored articles to be considered within the review) in decisions regarding inclusion or critical appraisal of their own work.	Reviewers who have authored articles to be considered within the review should be prevented from unduly influencing inclusion decisions, for example by delegating tasks appropriately.	Checklist	Yes	
29	Results (review findings)	Description of review process	Describe the review process including the volume of evidence identified from all sources and retained through each stage of the review. Must also display the number of articles/studies included at all stages of the review in a flow diagram, including the number of articles/studies excluded at each stage.		Checklist	Yes	
30		Number of search results	Provide the number of search results from bibliographic databases (including updates if conducted) prior to duplicate removal.	This number should not include web-based search engine or organisational website searches: this will help assessment of the efficiency of the primary search string.	Meta-data		26780
31		Number of search results after duplicate removal	Provide the total number of search results from bibliographic database searches following duplicate removal.	This number should not include web-based search engine or organisational website searches: this will help assessment of the efficiency of the primary search string.	Meta-data		20150
32		Full text screening excludes	Additional file containing list of and reasons for full text exclusions.		Checklist	Yes	
33		Title screening results	Provide the number of articles retained following title screening.	Optional if screening titles and abstracts together	Meta-data		6161
34		Abstract screening results	Provide the number of articles retained following abstract screening.	Optional if screening titles and abstracts together	Meta-data		2222
35		Title and abstract screening results	Provide the number of articles retained following title and abstract screening.	Optional if screening titles and abstracts separately	Meta-data	n/a	
36		Retrieval results	Provide the number of articles retrieved at full text.		Meta-data		2132
37		Unobtainable articles	Additional file containing list of unobtainable articles.		Checklist	Yes	
38		Full text screening results	Provide the number of articles retained following full text screening.		Meta-data		1230
39		Consistency checking: screening	Results of consistency checking at all stages (screening, meta-data extraction and coding, critical appraisal) must be provided. Provide the number of titles, abstracts and full texts screened and checked for consistency by two or more reviewers as a fraction of the total (e.g. Title: 2000/20000; Abstract: 500/5000: Full text: 10/100).		Checklist	Yes	

40		Narrative synthesis	Describe the body of evidence identified using figures and tables, avoiding vote-counting (tallying of studies based on results; direction or significance). Each must be presented with descriptive information (meta-data). Describe the validity of individual studies and the evidence base as a whole (if critical appraisal conducted).		Checklist	Yes
41		Systematic map database	Additional file containing meta-data and coding for included studies.		Checklist	Yes
42		Limitations of the review	Discuss possible limitations in the methods used.		Checklist	Yes
43		Limitations of the evidence base	Discuss possible limitations in the evidence base.		Checklist	Yes
44	Conclusions	Knowledge gaps and clusters	Describe knowledge gaps (unrepresented or underrepresented subtopics that warrant further primary research) and knowledge clusters (well-represented subtopics that are amenable to full synthesis via systematic review)		Checklist	Yes
45		Implications for policy/management	Summarise the state of the evidence base and discuss the way in which the identified evidence may inform policy/practice decision making in relation to the review/map question.	Reviews must not include practical environmental management recommendations or advocacy.	Checklist	Yes
46		Implications for research	Discuss the way in which the identified evidence may inform research including options for increasing the reliability of study design that could improve future research.	In this section some advocacy for future research on the reviewed topic is permissible provided it is clearly justified by the review outcome/critical appraisal of study validity.	Checklist	Yes
47	Declarations	Competing interests	Describe of any financial or non-financial competing interests that the review authors may have.		Checklist	Yes

References

[1] James, K.L., Randall, N.P. and Haddaway, N.R., 2016. A methodology for systematic mapping in environmental sciences. Environmental Evidence, 5(1), p.7.
[2] Bayliss, H.R., Haddaway, N.R., Eales, J., Frampton, G.K. and James, K.L., 2016. Updating and amending systematic reviews and systematic maps in environmental management. Environmental Evidence, 5(1), p.20.
[3] Haddaway, N.R., Kohl, C., da Silva, N.R., Schiemann, J., Spök, A., Stewart, R., Sweet, J.B. and Wilhelm, R., 2017. A framework for stakeholder engagement during systematic reviews and maps in environmental
[4] Collaboration for Environmental Evidence. 2018. Guidelines and Standards for Evidence synthesis in Environmental Management. Version 5.0. www.environmentalevidence.org/information-for-authors.
[5] Leeds Institute of Health Sciences. https://medhealth.leeds.ac.uk/info/639/information_specialists/1500/search_concept_tools. Accessed 12/11/2017.
